# Angiomyxoma of the ureter imitating an upper tract urothelial carcinoma: A case report

**DOI:** 10.1016/j.ijscr.2018.10.027

**Published:** 2018-10-19

**Authors:** Ahmed S. Zugail, Faris Baowaidan, Eva-Maria Comperat, Bertrand Guillonneau, Alexandre Colau

**Affiliations:** a Department of Urology, La Croix Saint Simon Hospital, Paris, France; b Department of Surgery, Faculty of Medicine in Rabigh, King Abdulaziz University, Saudi Arabia; c Department of Urology, King Faisal Medical City for Southern Region, Abha, Saudi Arabia; d Department of Pathology, Tenon Hospital, Paris, France

**Keywords:** Aggressive angiomyxoma, Angiomyxoma, Case report, Haematuria, Myxoid tumour, Ureter

## Abstract

**Introduction:**

Angiomyxomas are rare benign neoplasms of mesenchymal origin arising from the soft tissues of the perineum and pelvis.

**Case presentation:**

To our knowledge, we report the first case of ureteral angiomyxoma arising de novo in a fifty-four-year-old female patient who presented with macroscopic hematuria.

**Discussion:**

The pathological findings, radiological features and operative management will be discussed.

**Conclusion:**

Angiomyxomas are notorious for their locally infiltrative nature and their propensity to recur. Clinical suspicion is capital to reach the diagnosis and a holistic care is key to a good outcome.

## Highlights

• Angiomyxomas are rare benign neoplasms tumors arising from the soft tissues of the perineum and pelvis.
• Although benign, they are notorious for their locally infiltrative nature and their propensity to recur.
• To the best of our knowledge, we report the first case of ureteral angiomyxoma in the literature presenting with hematuria.
• If an angiomyxoma were to be considered in our differential diagnosis, a ureteral biopsy could have led to an organ-sparing surgery.

## 1 Introduction:

Steeper and Rosai [1] first described Angiomyxomas in 1983 but the term “*angiomyxoma*” dates back to at least 1952 by Raeburn. Angiomyxomas are rare benign mesenchymal stromal tumors that are remarkably slow growing. These tumors have a myxoid pattern and contain prominent angiomatous components characterized by large thick walled vessels without arborization. Due to their aggressive infiltrative nature and frequent recurrence behavior they are referred to as aggressive angiomyxomas or AAMs. They are generally found in the pelvis of adult females [2] but have been reported in males in very few cases. This work has been reported in line with the SCARE criteria [3].

## 2 Presentation of case:

**Fig. 1:**
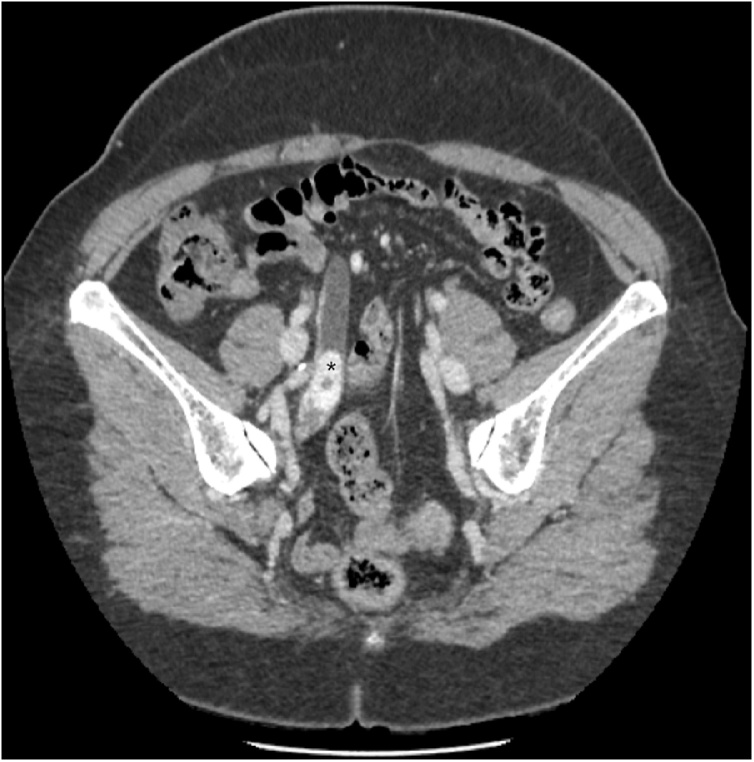
Oblique reconstruction late portal phase CT scan showing the tumor (asterisk) totally filling the right ureter.

**Fig. 2:**
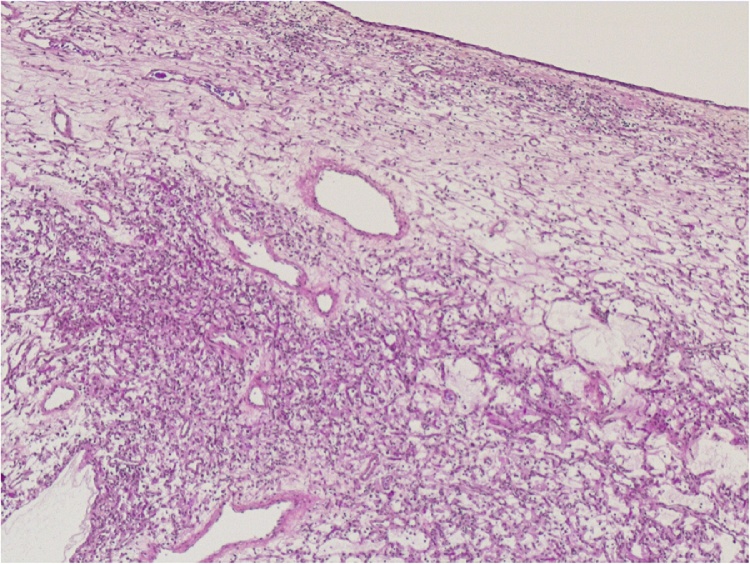
The urothelial tissue is represented as a thin layer on top. The lesion consists of multiple vessels and some connective tissue (hematoxylin phloxine saffron stain x 5).

**Fig. 3:**
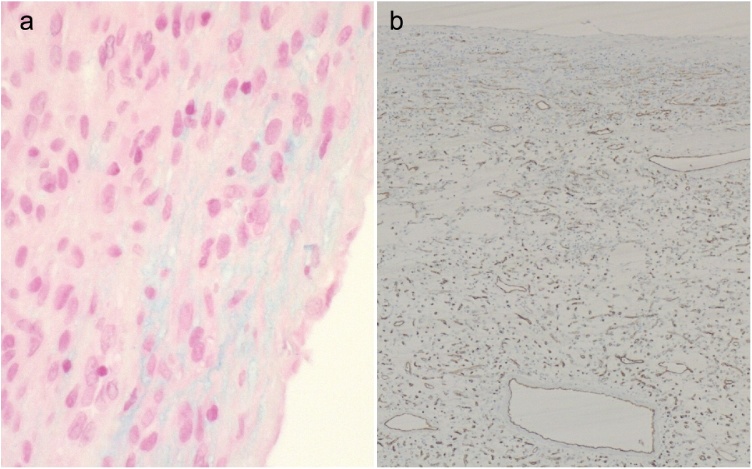
a. Alcian blue staining found in the interstitium. A typical feature of the collagenous structure of angiomyxomas (Alcian blue staining x 20). b. CD34 immunohistochemical staining highlighting the rich vascularization of the lesion (CD34 × 5).

We report a fifty-four-year-old overweight female patient (BMI 28.9) who came to the emergency department with a second episode of macroscopic hematuria. The first episode happened earlier after she fell on her back. Both episodes were spontaneously resolved after increasing oral intake of fluids. Clinical examination and blood work were unremarkable. She is known to have an idiopathic fascicular left ventricular tachycardia and a hypothyroidism that are well controlled by medication. The patient was referred to the urology outpatient clinic at our academic medical center with a uroCT (Computerized tomography) showing an obstructive worrisome lesion mimicking an upper tract urothelial carcinoma (UTUC) of the right pelvic ureter (Fig. 1). Furthermore, she underwent an office cystoscopy and a thoracic CT showing neither primary nor secondary lesions. The patient has no risk factors for urothelial cancer. After discussing the case in a multidisciplinary team meeting, a right nephroureterectomy with a right pelvic lymphadenectomy was proposed and done laparoscopically. The post-operative pathology report described a kidney measuring 11 × 8 cm and a ureter 20 cm in length. An endoluminal tumor of 3.5 cm of the ureter was shown macroscopically. Microscopically, the tumor showed stellate shaped fusiform cells with small monomorphic nuclei with fine chromatin, sometimes nucleolated. The lesion is richly vascularized with zones of small pseudo-cystic formations. The tumor is well limited and well housed by its thin fibrous capsule but is partially covered by normal ureteral tissue that testifies its position and development from the ureteral wall. Mitosis was non-existent. The tumor (Fig. 2) is heterogeneously positive with the alcian blue stain (Fig. 3a). The tumor is focally positive with smooth muscle actin. CD34 (Cluster of Differentiation) shows good vascularization (Fig. 3b) as well as factor 8. Cytokeratin 7 and CD68 are negative. AE1-AE3 (Anion Exchanger) and caldesmon are negative and desmin is weakly expressed. These findings suggested the diagnosis of an aggressive angiomyxoma. Ten malignancy-free lymph nodes were excised. Our resection is considered complete. A 12-month follow-up by CT scan showed no evidence of recurrent tumor.

## 3 Discussion:

Initially, these tumors were described as occurring only in women, although the female to male ratio is now estimated to be 6:1 [4]. The median incidence of this ailment is the fourth decade for women and the sixth decade for men. The youngest reported case was in a two-year-old boy. Localizations reported in females involve the vagina, vulva, uterine cavity and urinary bladder. In males, the scrotum spermatic cord, prostate, penis and urethra have been involved. To our knowledge, we report the first case report involving the ureter in the English and French literature. Distant metastases have been reported but are very rare [5]. AAMs should be distinguished from other benign tumors affecting the pelvis such as intramuscular myxomas, myxoid neurofibroma, myxoid or spindle cell lipoma, angiomyofibroblastoma and angiomyolipoma. The differential diagnosis of AAMs also includes some malignant tumors such as myxoid liposarcoma, myxoid malignant fibrous histiocytoma, and embryonal rhabdomyosarcoma. Imaging is capital for diagnosis and can characterize the mass and visualize the degree of extension into surrounding tissues prior to surgery. Ultrasound (US) imaging is not very helpful in the diagnosis of AAMs but can demonstrate the size, echogenicity, septations, solid components, and cystic structure of the mass. US-guided needle biopsy has been reported but was inconclusive. On computerized tomography (CT) scans, AAMs are hypodense or moderately enhanced delineated masses relative to muscles but still can have variable appearances. Magnetic resonance imaging (MRI) reveals a characteristic layered or swirling pattern seen in approximately 83% of cases making this modality more specific than CT [6]. Owing to the elevated water content and the loose matrix of these lesions, they are isointense relative to muscle on T1-weighted imaging, hyperintense on T2-weighted imaging and enhances avidly after the administration of gadolinium indicating its hypervascularity. Enhancement of the fibrovascular stroma unveils the characteristic layered or swirled appearance. AAMs are typically large centimetric lesions (1–60 cm). Macroscopic examination typically shows a non-capsulated, multilobular, soft, smooth, gelatinous, gray-white tissue with occasional firm fibrous areas. Microscopically, these tumors are bland with a hypocellular mesenchymal stroma. Cells are stellate or spindled in shape loosely scattered in the stroma with ill-defined cytoplasms. They’re rare or no evidence of nuclear atypia and mitotic figures. Rich blood vessels of varying sizes and types are normally present [7]. Immunohistochemical (IHC) features play a key role in the diagnosis of AAMs. Tumors usually are positive for vimentin, variably for muscle specific actin, α-smooth muscle actin and CD34, negative for desmin and S 100 protein [8]. These IHC tools distinguish this tumor from others previously mentioned. The majority of AAMs were reported in premenopausal females suggesting certain hormonal factors participating in the development or proliferation of AAMs. Also, the tumor was observed to be aggravated in pregnancy. Fittingly, it was found that greater than 90% of these tumors express estrogen (ER) and progesterone receptors (PR) and was found to be susceptible to hormone therapy in some patients [4]. Hormonal treatments with gonadotropin-releasing hormone (GnRH) agonists have been attempted but no data shows its effectiveness. Conversely, AAMs express androgen instead of estrogen receptors in some male cases [4]. Surgery with complete excision remains the first-line treatment for AAM eradication and incomplete resection can lead to recurrence. The highest recurrence risk reported was 72% and can be as close as six months from the initial resection but usually takes years. All recurrences reported were re-treated by surgery except for one that was treated by a GnRH agonist with complete resolution [9]. Positive surgical margins can occur due to soft tissue infiltration and the absence of a capsule. Surgical margins are not considered prognostic [10] but for most authors they remain the key predictive factor for recurrences. Chemotherapy is not indicated due to the no or low mitotic activity of the tumor. Other modalities of management failed to treat AAMs such as radiotherapy due to the radioresistant nature of the tumor seen after some trials. Another example of failed treatment is angio-embolization owing to the tumor’s rich vascularization [10]. Patients with AAMs require close follow-up after resection to monitor recurrence. There is no consensus concerning the duration and nature of the follow-up, however most physicians use a cross-sectional imaging modality, usually an MRI. We believe that if an angiomyxoma were to be considered in our differential diagnosis, a ureteral biopsy could have led to an organ-sparing surgery.

## 4 Conclusion:

AAMs are rare benign neoplasms that are more predominant in females. Though rare, clinical suspicion is required in order to reach the diagnosis, and a multidisciplinary care is key to obtaining a good outcome. A careful primary surgical extirpation, as complete as technically feasible, should be the main primary surgical approach. The importance of pre-operative biopsy in this case should be mentioned. Local recurrence is common. The role of GnRH agonist treatment is unclear. There is no standard follow up regimen for AMMs though it should include an imaging modality.

## Conflicts of interest:

None.

## Sources of funding:

None.

## Ethical approval:

Not applicable. The study is exempt from ethical approval in our institution.

## Consent:

Written informed consent was obtained from the patient for publication of this case report and accompanying images. A copy of the written consent is available for review by the Editor-in-Chief of this journal on request.

## Author contributions:

Ahmed S. Zugail: Study concept, data collection and interpretation, writing the paper.

Faris Baowaidan: Study concept, data collection and interpretation, writing the paper.

Eva-Maria Comperat: Study concept, data collection and interpretation, writing the paper and supervision.

Bertrand Guillonneau: Study concept, data collection and interpretation, writing the paper and supervision.

Alexandre Colau: Study concept, data collection and interpretation, writing the paper and supervision.

## Registration of research studies:

Not applicable.

## Guarantor:

Ahmed S. Zugail.

## Provenance and peer review

Not commissioned, externally peer reviewed.
